# Upgrade of a semi-automatic flow injection analysis system to a fully automatic one by means of a resident program

**DOI:** 10.1155/S1463924695000289

**Published:** 1995

**Authors:** M. I. Prodromidis, A. B. Tsibiris, M. I. Karayannis

**Affiliations:** Laboratory of Analytical Chemistry Department of Chemistry University of Ioannina University Campus Ioannina 45110 Greece

## Abstract

The program and the arrangement for a versatile, computer-controlled flow injection analysis system is described. A resident program (which can be run simultaneously and complementary to any other program) controls (on/off, speed, direction) a pump and a pneumatic valve (emptying and filling position). The system was designed to be simple and flexible for both research and routine work.


Journal of Automatic Chemistry, Vol. 17, No. 5 (September-October 1995), pp. 187-190

### Upgrade of a semi-automatic flow injection analysis system to a fully automatic one by means of a resident program

**M. I. Prodromidis, A. B. Tsibiris and M. I. Karayannis**

*Laboratory of Analytical Chemistry, Department of Chemistry, University of Ioannina, University Campus 45110, Ioannina, Greece*

*The program and the arrangement for a versatile, computer-controlled flow injection analysis system is described. A resident program (which can be run simultaneously and complementary to any other program) controls (on/off, speed, direction) a pump and a pneumatic valve (emptying and filling position). The system was designed to be simple and flexible for both research and routine work.*

### Introduction

Since the first report on the microprocessor control of an FIA device [1], numerous papers have been published describing different aspects of FIA control [2-5]. The majority of the automated instruments available are coupled with powerful software systems, which require the complete use of a personal computer as a set of switchers. Resident (TSR) programs in order to improve the performance of an instrument (for example, control of a multi-channel gas valve and simultaneously monitoring of the gas pressure inside a catalytic reactor or to upgrade a manifold as with the work being carried out here) would be attractive to most users. A TSR program automates control using the computer already in place and optimally synchronizes with the software for data acquisition (for example, time dependent temperature and humidity control of a gas-sensing amperometric cell, sampler in an FI manifold, etc.).

The software package presented here provides the best working conditions for laboratories working with FIA, because it is compatible with all detector types (photometric, fluorometric, potentiometric, amperometric, etc.). With this package a semi-automatic system can be fully automated and will be capable of operating for some hours (in particular cases, for some days) without supervision.

All of the electrochemical experiments used to test this software package were performed on an Autolab Electrochemical Analyser with the 'General Purpose Electrochemical System' (GPES3) software (Eco chemie BV, Utrecht, The Netherlands). This system is capable of collecting and evaluating data provided by the detector and can produce on-line graphs.

A TSR like the one presented in this work can be run with the GPES3 or with any commercial or individual program. The full control of the pump and the injection valve gives to the user the ability: (1) to work without manual manipulations; (2) to repeat an experiment many times in order to improve statistical parameters (average, relative standard deviation); and (3) to measure different sample volumes during a 'simplex optimization' experiment, with high accuracy and reproducibility without replacing the loop. The full pump control allows also a good enzyme immobilization, by circulation of the enzyme solution through a reactor bearing the support, for any desired period and both flow directions.

Correspondence to Professor Karayannis.

### Design

*Apparatus*

The FIA manifold is shown in figure 1. A Gilson Minipuls 3, four-channel (Middleton, USA) peristaltic pump was used. The system also includes a four-way pneumatic injection valve (Rheodyde 5701, California, USA) linked with a home made electronic actuator (see figure 2) consisting of a DC transformer (5 to 12 V, 2 A, 5 W) connected to a 12 V coil. The coil can be actuated by the software (5 V in output), allowing the compressed air to pass through the valve. The circuit for the valve control is very simple and inexpensive. A three-electrode electrochemical detector (Metrohm, model 656, Herisau, Switzerland) was used. It comprises a wall-jet type thermostatted cell (volume <1 μl), a working electrode (graphite, 3·0 mm i.d., Ringsdorff, Germany), a Ag/AgCl reference

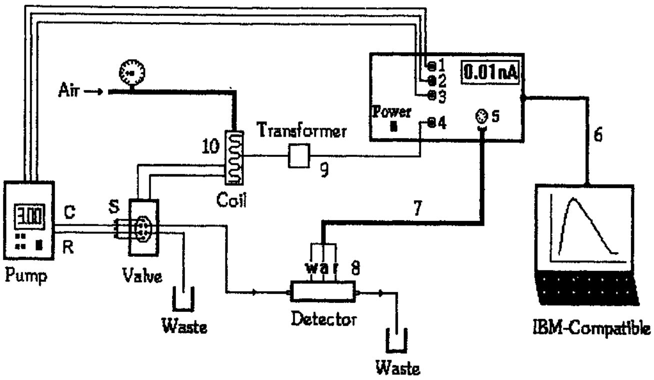

*Figure 1. Flow injection analysis manifold: 1, 2, 3, 4—digital to analogue converters with a resolution of 16 bits. 5—DIN connnector. 6—Interface cable. 7—The cell cable with four connectors for the working (w), reference (r), auxiliary (a) electrodes and the analogue ground. 8—Electrochemical detector, with a wall-jet type thermostatted cell (volume <1 μl). 9—DC transformer. 10—12 V coil (selenoid). C—carrier; R—reagent; S—sample.*

0142-0453/95 $10.00 © 1995 Taylor & Francis Ltd.

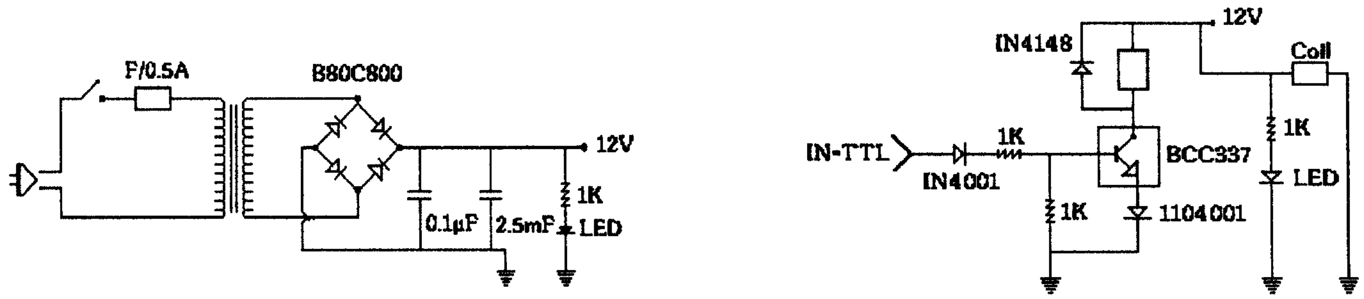

*Figure 2. Home-made electronic actuator for the activation of the valve.*

electrode and a built-in auxiliary electrode (Au). The potentiostat is the Autolab Electrochemical Analyser. PVC tubes (0·50 and 0·38 mm i.d.) were used for the transportation of the carrier and the reagent streams, respectively.

### *Hardware*

The program can operate with any type of processor, including the 8086. In this work a IBM compatible computer was used. The Autolab [6] is a modular system. The standard modules are: ACD 124, DIO 48 and DAC 164.

The DAC164 module produces an analogue output as specified by a digital code of 0s and 1s with a resolution of 16 bits or 300 μV and a range of $\pm 10$ V. The DAC 164 provides four channels of analogue outputs. The limit of the output current of the DAC 164 unit is at least $\pm 20$ mA. The setting time for a 20 V full-scale transition to 0.01% precision is normally 3·5 μs. The output of the converter is minus full scale when 0 and plus full scale when 65536 is sent. This module requires three I/O-ports. The hexadecimal addresses are [6]: 280H: select DAC, 281H: low byte of DAC, 282H: high byte of DAC.

### *Software*

First the proper DAC must be selected, by sending a byte to I/O-port 280H. This byte is: DAC 1: OEFH, DAC 2: ODFH, DAC 3: OBFH, DAC 4: O7FH. All DACs are disabled when OFFH is sent to I/O-port 280H. The DAC can be set at a specified output voltage, by first sending the low byte to I/O-address 281H and then by sending the high byte to I/O-address 282H. Since the DAC has a resolution of 16 bits, the 16-bits word sent to the DAC must be in the range 0 to 65536 (OH to FFFFH) [6].

A menu-driven program (see figure 3) was developed using Turbo Pascal, Version 5·5 (Borland Corp., CA, USA). It contains two subprograms. With the first one, (NTSR) introduces the settings for the pump and valve control (start/end time, speed, direction, function). The valve setting menu (figure 3) is activated by choosing the 'Experimental mode'. When the 'Immobilization mode' is chosen the 'Experimental mode' submenu is deactivated. This program needs 14 KB of memory. All the settings of this subprogram are saved in a file. The TSR subprogram occupies 12 KB of memory. It is loaded into the RAM and controls the FIA components up to the time lapse or when the combination 'Alt-R Shift' (see below) is pressed; 2048 byte of the TSR program are allocated to the values of the applied variables.

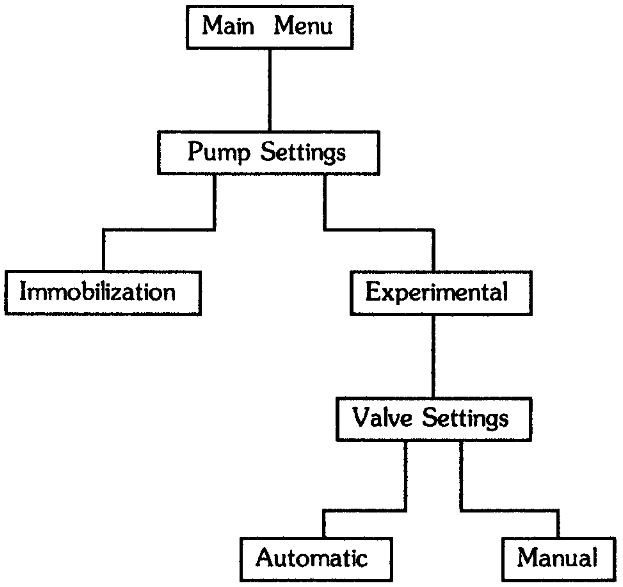

*Figure 3. Dendrogram of the AutoFIA program showing the submenu options.*

*Table 1. Connections of the DAC 164 with the valve and the pump. Port 1 for valve control, port 2 for pump switch, port 3 for pump direction and port 4 for pump speed.*

| DAC 164 Ports | Valve 0 V | Valve 5 V | Pump 0 V | Pump 5 V |
|---|---|---|---|---|
| 1 | Filling position | Emptying position | — | |
| 2 | — | | On/pin4 | Off/pin3 |
| 3 | — | | Left/pin2 | Right/pin1 |
| 4 | — | | 0–5 V, giving 0–100% of the selected speed/pin5,6 | |

The activation and full control of the FIA components is achieved by means of eight procedures: turn on/off valve (filling position/emptying position), turn on/off pump, pump speed, turn pump right/left and turn off all. For each procedure the proper port of the DAC 164 (see table 1) is selected first, which entails the transformation of voltage values to a low and a high byte [6].

The most complicated of these procedures is pump speed control, where, in addition to the transformation described above, it also transforms voltage to rpm:

```
Procedure Pump Speed;
Var Spdstep: Real;
  DACval: Integer;
  IDAC          , IPORTDACSEL ,
  IPORTDAC_L          , IPORTDAC_H ,
  IDACVALUE , IDACVALUE_H ,
  IDACVALUE_L             : Integer;
Begin
  Spdstep := 341.33;
  DACval := ROUND(INT(Spdstep * Pspd));
  IDACVALUE_H := ROUND(INT(DACVAL/256));
  IDACVALUE_L := ROUND(INT(DACVAL - 256
  * IDACVALUE_H));
  IDAC              := $07F;
  IPORTDACSEL          := $280;
  IPORTDAC_L           := $281;
  IPORTDAC_H           := $282;
  PORT[IPORTDACSEL] := IDAC;
  PORT[IPORTDAC_L] := IDACVALUE_L;
  PORT[IPORTDAC_H] := IDACVALUE_H;
End;
```

The keys on the keyboard are inactive since the read key commands are out of operation. The deactivation of the TSR program and the on-line activation of the valve (Experimental/Manual) is achieved with the combination of the 'Alt-R Shift' and 'Ctrl-R Shift' respectively [7], through the BIOS using the command MEM [$0040:$0017].

In the Experimental/Automatic mode a number of up to 20 time settings can be chosen for the operation of the valve at the filling or the emptying position. The ability allows the repetition of an experiment (different sample size, different sample concentration, the latter when a sampler is available) and provides the most reproducible experimental conditions (see figure 4). In the Experimental/Automatic mode, on-line activation of the valve with the combination of 'Ctrl – R Shift' is also possible.

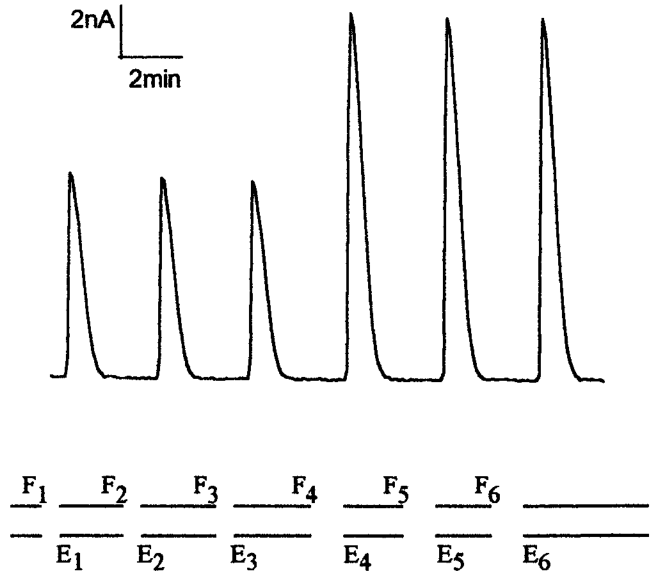

*Figure 4. FIA recordings obtained automatically for different sample size (30 µl and 60 µl). The time settings in experimental/automatic mode were: $F_1 = 10$, $E_1 = 20$, $F_2 = 170$, $E_2 = 180$, $F_3 = 330$, $E_3 = 340$, $F_4 = 490$, $E_4 = 510$, $F_5 = 660$, $E_5 = 680$ and $F_6 = 830$, $E_6 = 850$. F, filling position and E, emptying position. Time in seconds.*

In order to obtain the best synchronization between the TSR and the acquisition software the activation and deactivation of the time settings can be achieved with the combination of 'R Shift – Insert':

```
If Mem[$0040:$0017] and $09 = $09 Then Begin {Alt
– R Shift}
  Close_Pump       ;
  Close_All     ;
  SetIntVect($1C, Vector);
  Clrint        ;
End;
If Mem[$0040:$0017] and $05 = $05 Then Begin {Ctrl_R
Shift}
  If ((Pact = 'E') or (Pact = 'X')) Then Begin
        Turn_Valve ;
  End;
If Mem[$0040:$0017] and $80 = $80 Then Begin {R
Shift – Insert}
Fla := 1;
End;
```

The 'GetIntVec' command blocks the location $1C of the RAM and then stores it at the variable Vector. At this location of the RAM the procedure 'TestHotKey' is introduced using the Turbo Pascal [10] command 'GetIntVec'.

The 'TestHotKey' procedure uses the registers of the microprocessor which control the data and execute several tasks. The registers are incorporated in the processor at specified store locations of 16 bit:

```
Procedure TestHotKey(Flags,CS,IP,AX,BX,CX,DX,SI,
DI,DS,ES,BP :word);
interupt;
var ch              : char;
  memor : word;
    IntPointer   : Pointer;
    M1, S1              : Longint;
    H1, Days    : word;
Begin
––––––Commands–––––
End;
```

### System performance

All the test experiments were performed using a Glycerol dehydrogenase (GDH, E.C1.1.1.6, 49 IUnits·mg$^{-1}$, Sigma) reactor [9]. The reactor contains 80 mg support and has the dimensions 2 mm i.d. and 30 mm length. Inside the reactor the CPG beads were ordered with a specific orientation, depending on the direction of the flow.

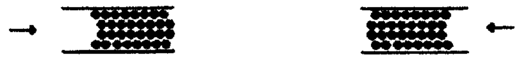

*Figure 5. Glass beads orientation at different flow directions.*

The periodical change of the flow direction enhances the immobilization efficiency. The GDH solution (7 mg GDH in 3·5 ml phosphate $5 \times 10^{-2}$ M, pH = 8) was continuously pumped through the reactor for 36 h and the direction was changed every 500 s. Under these conditions a 3–8% increase in immobilization efficiency can be achieved.

The sample loop has a total capacity of 180 µl. All measurements were carried out at an applied potential +0·5 V versus a Ag/AgCl reference electrode. A Tris-HCl buffer solution 50 mM, pH 9, was used as the carrier stream, and a $NAD^+$ solution 4·5 mM in the buffer solution as the reagent stream. The applied flow rates were 0·18 and 0·12 $ml \cdot min^{-1}$ for the carrier and the $NAD^+$ streams, respectively.

At this flow rate (0·18 $ml \cdot min^{-1}$) the loop is filled in 60 s, which means that 3 µl are transferred each second. For example, if a volume of 60 µl is needed, the valve is programmed to turn at the filling position for 20 s of operation. The software was applied in a Simplex Optimization experiment where the sample size is one of the most crucial parameters. All the sample volumes in the range 21–360 µl, were measured, using the same loop, by propelling solutions at a 3 µl step with high accuracy and reproducibility.

Using the proposed software the relative standard deviation (r.s.d., %) of the method was significantly improved, because the manual manipulations of the valve, even by complete filling of the loop, introduces errors. For total of 50 injections (10 series of five injections) of a 0·05 mM glycerol (150 µl sample size), the total %RSD was 0·72%.

### Conclusions

The resident program described is a very versatile, cheap and easy-to-operate software. Programs of this type are useful in every laboratory because a semi-automatic FIA manifold (data acquisition and evaluation) can be upgraded to an automatic one. Pump control is a powerful tool especially in the case of enzyme immobilization where the direction of the flow must be altered periodically every 10–20 min for a period of 12–72 h [9]. This software offers high immobilization efficiencies which could not be realized manually. Valve control is also very powerful for any kind of FIA experiment (routine, relative standard deviation, Simplex Optimization), because it allows the selection of different sample volumes with high accuracy and reproducibility, without replacing the loop. The electric actuation of the pneumatic valve with a transformer and the coil is also a very simple and cheap construction.

### Acknowledgements

The authors wish to thank Mr A. Antoniou (†RIP) for constructing the electromechanic components. Thanks are also extended to Dr G. Brug and Mr A. Schild from Eco Chemie BV for valuable advice during the development of this system and to Professor C. E. Efstathiou for his useful comments.

### References

1. Stewart, K. K., Brown, J. F. and Golden, B. M., *Analytica Chimica Acta*, **114** (1980), 119.
2. Clark, G. D., Christian, G. D., Ruzicka, J., Anderson, G. F. and van Zee, J. A., *Analytical Instrumentaion*, **18** (1989), 1.
3. Marshall, G. D. and van Staden, J. F., *Analytical Instrumentation*, **20** (1992), 79.
4. Koupparis, M. and Anagnostopoulou, P., *Journal of Automatic Chemistry*, **6** (1984), 186.
5. Keller, J. W., Gould, T. F. and Aubert, K. T., *Journal of Chemical Education*, **63** (1986), 709.
6. *Autolab Installation and Operation Guide*, Eco Chemie B.V., Utrecht, The Netherlands (1991).
7. Wyatt, A. L., Sr., *Using Assembly Language*, Que Corp., USA (1990).
8. Wood, S., *Using Turbo Pascal*, Borland-Osborne, McGraw-Hill Inc., N.Y., USA (1989).
9. Prodromidis, M. I., Stalikas, C. D., Tzouwara-Karayanni, S. M. and Karayannis, M. I., Unpublished results.

